# Haplotypes for Type, Degree, and Rate of Marbling in Cattle Are Syntenic with Human Muscular Dystrophy

**DOI:** 10.1155/2017/6532837

**Published:** 2017-08-17

**Authors:** Sally S. Lloyd, Edward J. Steele, Jose L. Valenzuela, Roger L. Dawkins

**Affiliations:** ^1^CY O'Connor ERADE Village Foundation, P.O. Box 5100, Canning Vale South, WA 6155, Australia; ^2^Melaleuka Stud, 24 Genomics Rise, Piara Waters, WA 6112, Australia; ^3^Centre for Innovation in Agriculture, Murdoch University, Murdoch, WA 6150, Australia

## Abstract

Traditional analyses of a QTL on Bota 19 implicate a surfeit of candidates, but each is of marginal significance in explaining the deposition of healthy, low melting temperature fat within marbled muscle of Wagyu cattle. As an alternative approach, we have used genomic, multigenerational segregation to identify 14 conserved, ancestral 20 Mb haplotypes. These determine the degree and rate of marbling in Wagyu and other breeds of cattle. The melting temperature of intramuscular fat is highly heritable and traceable by haplotyping. Fortunately, for the production of healthy beef, some of these haplotypes are sufficiently penetrant to be expressed in heterozygous crossbreds, thereby allowing selection of sires which will improve the healthiness of beef produced under even harsh climatic conditions. The region of Bota 19 is syntenic to a region of Hosa 17 known to be important in muscle metabolism and in determining susceptibility to a form of human muscular dystrophy.

## 1. Introduction

Conserved ancestral haplotypes have been highly informative in revealing the functional significance of polymorphic sequences especially when extensive enough to encode regulatory regions as well as structural elements [1–3].

In *Homo sapiens* (Hosa), there are now many examples of conserved haplotypes regulating the degree of expression of a trait or disease such as an enzyme deficiency, immune competence, drug reactions, and disease severity. The best known examples are the extended haplotypes of the major histocompatibility complex [1, 2, 4–7], but the principle applies throughout the genome, [8] with the important caveat that the haplotypes are identified and tagged correctly [9, 10]. Many of these haplotypes were discovered through the finding of genetic associations with quantitative traits, such as diseases. It became obvious that there are tens or even hundreds of alternative haplotypes encoding functionally important regions of the genome and that these interact with each other positively or negatively. A potent example is IgA deficiency [2, 6, 11–13] which is associated with the 8.1 haplotype *inter alia*. The precise mechanisms underlying this regulatory defect are not clear from analysis of SNPs and coding sequences [14].

For the breeding of livestock, it is traditional [15, 16] to follow the precepts of the infinitesimal model which assumes there are independent genes separated by free recombination [17]. However, it is quite clear that this assumption is not correct [3, 17]. The genomic architecture of cattle [18–21], sheep [22, 23], goats [24], dogs [25, 26], mice [27], and pigs [28, 29] is remarkably similar to that present in Hosa, where there are kilobase and even megabase blocks of polymorphism at which there are retroviral and other indels, segmental duplications, and differences in copy number in addition to nucleotide substitutions [1, 2, 30, 31].

In cattle, there are many traits of proven importance to the production of healthy food. There have been numerous attempts to find useful genetic markers if only for selection of superior breeding pairs for commercial gain. Results have been disappointing for several reasons generally reflecting an oversimplified understanding of the genomic structure. Thus, conserved ancestral haplotypes can be confounding if not identified. For example, desirable traits such as increased milk production can be associated with disadvantages such as infertility [3]. In other instances, the genetic marker may appear useful in one breed but not in another, as illustrated by polling [3].

Here, we address these disappointments by identifying megabase haplotypes of critical interest to healthy food and public health [32, 33] and potential relevance to the metabolic changes underlying limb girdle muscular dystrophy.

True or fine marbling describes the deposition of lipid between and within muscle fibres after supplementary feeding for a year or more occurs. The genesis is very complex, including differentiation of satellite or progenitor cells, and is obviously tightly regulated, [34], but the ultimate effect is to improve taste and juiciness thereby increasing the value of beef several folds. More importantly, marbling increases the content of the healthy, monounsaturated fatty acid (MUFA) and is therefore “cholesterol lowering” and statin-sparing [35, 36]. These features are characteristic of Japanese cattle, known as Wagyu, black and red, but may be found to lesser degrees in other breeds and crossbreeds. It is important to distinguish true or fine marbling from the deposition of subcutaneous and interfascicular fat as a characteristic of European breeds.

For more than 20 years, initially in Japan, there have been attempts to find predictors of

- how much a given animal will marble;
- which matings are preferable;
- whether the very costly feeding can be reduced.

At face value, the results are contradictory but also tantalising since the inconsistencies may be due to the complexity of the marbling pathway and the sheer number of genes involved.

A simple polymorphism of the SCD (delta-9 desaturase on Bota 26) appears to affect the amount of marbling presumably by encouraging desaturation and therefore the production of MUFA and particularly oleic acid. Polymorphisms of growth hormone (GH) and fatty acid synthase (FASN) amongst many others may also have an effect. The evidence for some of these individual genes is reviewed and presented in Table 1 [37–54]. From these 14 studies, the only fair conclusion is that, although the genomic region controls fat deposition, multiple genes and therefore haplotypes must be involved.

Our interest increased when it became apparent that a regulator of SCD, SREBF1, maps to the Bota 19 chromosome together with GH and FASN. Taken with the pleiotropic regulatory role of SREBF1, in humans at least [55], and with the evidence for the influence of Bota 19 on fatty acid composition in Japanese black cattle [48, 49], we asked whether extensive, multimegabase haplotypes could be identified. Previous investigations [37–54] have suggested that some intervening and adjacent genes could be relevant. Such apparent clustering of plausible candidates is often illusory, but in this case, we were encouraged by the fact that Japanese breeders are sufficiently convinced to be prepared to pay handsomely for SCD and GH typing even though it seems clear that these genes are unlikely to be involved directly.

Accordingly, we have been defining conserved ancestral haplotypes within the region. The many conserved megabase haplotypes have very different frequencies when breeds are compared [56]. The need for a simple summary of breed differences led to the development of the W plots which separate haplotypes common to all cattle from those which are found in, and potentially defining for, one breed but not in the other [57, 58]. Of course, since Wagyu cattle marble, it follows that all Wagyu-specific haplotypes must be associated with marbling irrespective of their function; all too often, such passive associations have been misinterpreted as causal. Common haplotypes may be implicated unless nonmarbling breeds are compared to determine which are specific for marbling breeds. The challenge is to assess the Wagyu haplotypes in different backgrounds such as can be obtained by crossing Wagyu with other breeds which are relatively resistant to marbling.

As a strategy, we asked whether Wagyu-specific haplotypes, once defined, are penetrant when heterozygous in F1 crossbreds. If so, it would be fair to conclude that these haplotypes have direct effects on the marbling process. We also asked whether non-Wagyu haplotypes affect the *rate* of marbling alone or in combination. Therefore, we started the investigation by determining whether fine marbling of progeny is a function of parentage.

In order to undertake such studies, it is critical to have a reliable measurement of marbling. In spite of many attempts, including the use of photographic and ultrasound images, there is still no agreed international benchmark, largely because the deposition of the preferred fat is very fine and barely visible. The most objective approach is the cumbersome slip test for the measurement of melting temperature (*T*_m_) of the lipid fraction and effectively the predominance of oleic acid [34, 59]. In Wagyu, *T*_m_s below body temperature (37°C) correlate with fine marbling thereby explaining the cholesterol lowering and statin-sparing effects [34].

We have introduced a more convenient and highly reproducible variation using the same thermocycler as used for the polymerase chain reaction [60].

With this method, elsewhere we will show that low *T*_m_ is highly heritable as expected given its extreme expression in only the Wagyu breed. Furthermore, we show that the effect is determined by multiple, conserved, megabase haplotypes. These can be used to predict marbling in crossbreds. There is evidence for *cis*-interactions within and *trans*-interactions between haplotypes. We also describe a strategy for improving the quality and healthiness of beef by judicious crossing.

The step-wise approach used here can be applied to other challenges in livestock genetics especially where regulatory ancestral haplotypes are suspected. Systematically, we address

- synteny as a guide to functions and interactions already defined in Hosa;
- breed effects as an initial screen for heritability;
- heritability as the only variable;
- genomic markers of inheritance;
- haplotypes in crossbreds;
- mechanisms of haplotype effects.

The conclusions of all these complementary studies lead to a model of the genetic control of fatty acid composition.

## 2. Results

### 2.1. Synteny as a Guide

**Figure 1:**
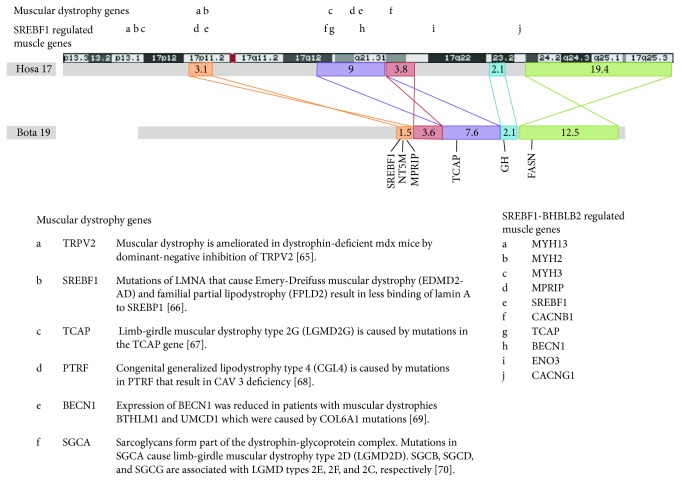
Marbling and muscular dystrophy are syntenic on Bota 19 and chromosome 17. Coloured boxes represent segments with the same gene content. Crossed joining lines indicate inverted translocations. Numbers represent Mb. Synteny was determined by the positions of homologous genes in the human assembly Hg 38 and bovine assembly BosTau8 located using the UCSC Genome Browser. Inverted sections and the exact location of boundaries between blocks were determined by dotplots [63] comparing the two sequences. The annotated dotplots used are shown in Supplementary Figure 1 available online at https://doi.org/10.1155/2017/6532837. The positions of genes associated with muscular dystrophy are shown in the first row of letters above Hosa 17. The association to muscular dystrophy is shown in the table below [65–70]. The positions of genes involved in the regulation of muscle development by SREBF1, either directly or through BHLHE40 and BHLHE41 (previously known as BHLHB2 and BHLHB3, resp.), are shown in the second row of letters above Hosa 17. Adapted from [3] with permission. We thank Dr. Joe Williamson for the assistance with this figure.

In Figure 1, we show the regions of Hosa chr17 which correspond to SREBF1 to FASN on Bota chr 19. Note also the regulatory effects of SREBF1 on remote sites many of which map closely with locations implicated in limb girdle muscular dystrophy.

In the dot plot of GH to FASN, it is apparent that there are two separate components in Hosa; the blue and green sections are distinct but note how they remain contiguous even though the green has been inverted. Similar phenomena are present elsewhere in the synteny plot. Together, these suggest that there are multiple interacting haplotypes from SREBF1 to FASN.

### 2.2. Breed Effects and Heritability

Most Wagyu have *T*_m_ of intramuscular fat below human body temperature of 37°C and distinctly lower than fat from Simmentals which is mostly subcutaneous and generally above 40°C [60]. The length of feeding was much greater for Wagyu due to the fact that the Simmentals grow rapidly and fatten quickly through deposition of largely subcutaneous fat. Wagyu are much slower to both grow and fatten as they deposit intramuscular rather than subcutaneous fat.

We compared Wagyu cattle with various European breeds and crossbreeds which were fed the same ration under the same environmental conditions, but with days on feed varied to achieve the desired finish. The results showed that the *T*_m_ of the subcutaneous fat was lower in animals with higher proportions of Wagyu ancestry (data not shown here). Differences were noticeable in animals with only 25% Wagyu ancestry indicating that the breed effect can be highly penetrant.

Wagyu steers were fed together for 300 days and assessed identically. The only significant variable was parentage which was confirmed by DNA haplotyping. All sires are considered to be elite in terms of their breeding, reputation, and estimated breeding value (EBV) when available. The progeny groups differ significantly, with mean *T*_m_ for the progeny of two sires differing by nearly 2 degrees. Importantly, this effect was not seen with the visual marbling score. There is an unequivocal genetic effect detectable within one generation. Thus, in spite of the undoubted complexity of the inheritance, sires can be ranked according to heritability of low *T*_m_.

Thus, breed differences, individual sires, and presumably identifiable genetic factors, determine the type, rate, and location of lipid deposition. However, it is also clear that there is a major effect of environment in that *T*_m_ falls with increasing DOF irrespective of breed, and this fact compounded by imprecision may have contributed to past confusion.

In the analysis of genetic differences presented here, we define alleles and haplotypes common in Wagyu but rare in other breeds that may explain some of the differences in the type, rate, and location of lipid deposition.

### 2.3. Genomic Markers of Inheritance

**Figure 2:**
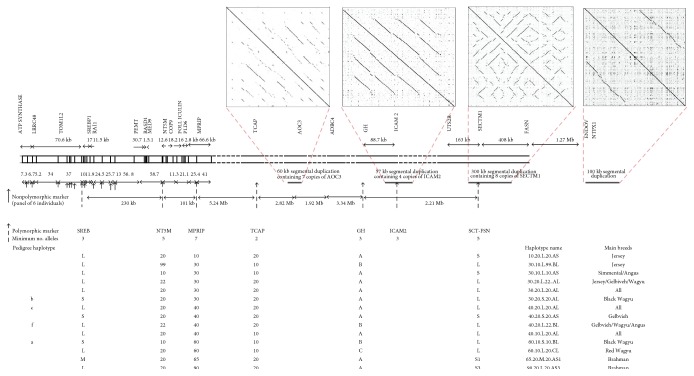
Haplotypes of the marbling region. Polymorphic markers define conserved extended C19 ancestral haplotypes: This region on BTAchr19 is bounded by markers SREBF1 and FASN (see [56] for details). The FASN marker is more correctly known as SCT-FSN since the present marker is adjacent to the coding region for FASN, within the segmental duplication containing SECTM1. The map positions of PCR markers and the number and type of alleles at each locus are indicated including the main high frequency and largely breed-specific haplotypes extending from SREBF1 to TCAP. We have extended the haplotyping through the region and plan to develop more markers based on the potential polymorphism revealed by the structural duplications extending from 43 Mb to 52 Mb (figure and below). Note the regions where PCR product polymorphism was not detected [56]. In structural duplications in C19 35-55 Mb region, we used the current cow genome assembly (Bos_tauros_UMD_3.1.1/bostau8 Assembly) on the UCSC Web Browser and searched the chr19 region from 35 Mb to 55 Mb in 500 kb sectors for large structural segmental duplications using standard dot-plotting methods aligning each 500 kb sector against itself (Gepard 1.30) [63]. We found clusters of rolling, sometimes clustered and inverted, segmental duplications in the reference genome on chr19 at 43.51 Mb (~60 kb in length), 43.86 Mb (~90 kb), 48.86 (~57 kb), and 50.846 Mb (~300 kb). We also found a long single imperfect duplication of 103–112 kb at 52.73 Mb and 52.88 Mb. Some of these dot plots are shown as cutaways in the figure. This region has a relatively low density of protein coding genes.

The genomic structure of the relevant region of Bota 19 contains the previously implicated SREBF1 and GH, as shown in Figures 1 and 2. Note the extensive duplication as found in other genomic regions of polymorphism including differences in copy number. The degree of polymorphism (at the insensitive gel level) ranges from at least 7 alleles at the most polymorphic locus (MPRIP) to only 2 at the least (TCAP). In the SREBF1 to GH (S-G) segment, there are 3 × 5 × 7 × 2 × 3 = 630 possible haplotypes on each paternal or maternal chromosome. Therefore, there should be some thousands of genotypes if the alleles at each locus are segregating randomly due to free recombination as specified in the infinitesimal model of population genetics.

**Figure 3:**
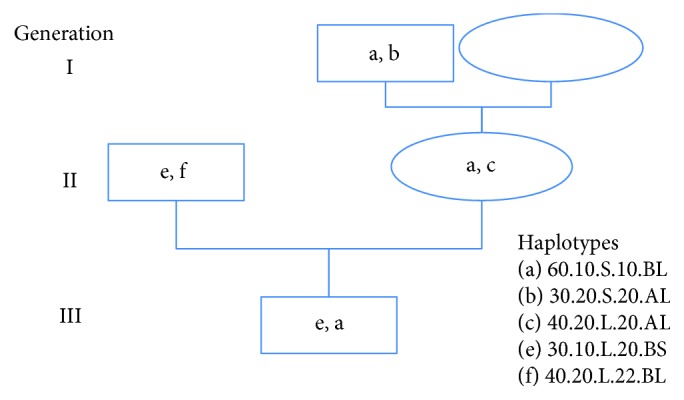
Segregation of 20 Mb haplotype through three generations of Wagyu pedigree. Haplotype (a) is an inherited intact from the maternal grandsire. The calf, dam, and maternal grandsire are all heterozygous at the SREBF1 or NT5M and GH or FASN markers, which allows the segregation of haplotype (a) to be seen clearly.

Here, we report the actual frequencies in Wagyu and in some of the breeds with which they have been crossed. Only 14 haplotypes occur commonly, say greater than 5%, in the breeds studied here. Importantly, as shown in Figures 3() and 4(), these are the conserved ancestral haplotypes. Four of the fourteen are present in the three-generation family which also shows the segregation of the (a) haplotype in three genotypes. None of the haplotypes could be identified through linkage disequilibrium; they must be observed empirically. They are *not* recent recombinants or mutants. They are *not* those expected from the allele frequencies and random assortment. Random segregation and free recombination can be excluded at least in this 20-megabase region.

As shown in Figure 2, haplotypes are designated by the alleles at MPRIP, TCAP, SREBF1, and NT5M in that order so as to emphasize the conservation over the 5 megabases between MPRIP and TCAP (M-T segment). Note in Figure 1 that MPRIP and TCAP are adjacent in Bota but not in Hosa.

**Figure 4:**
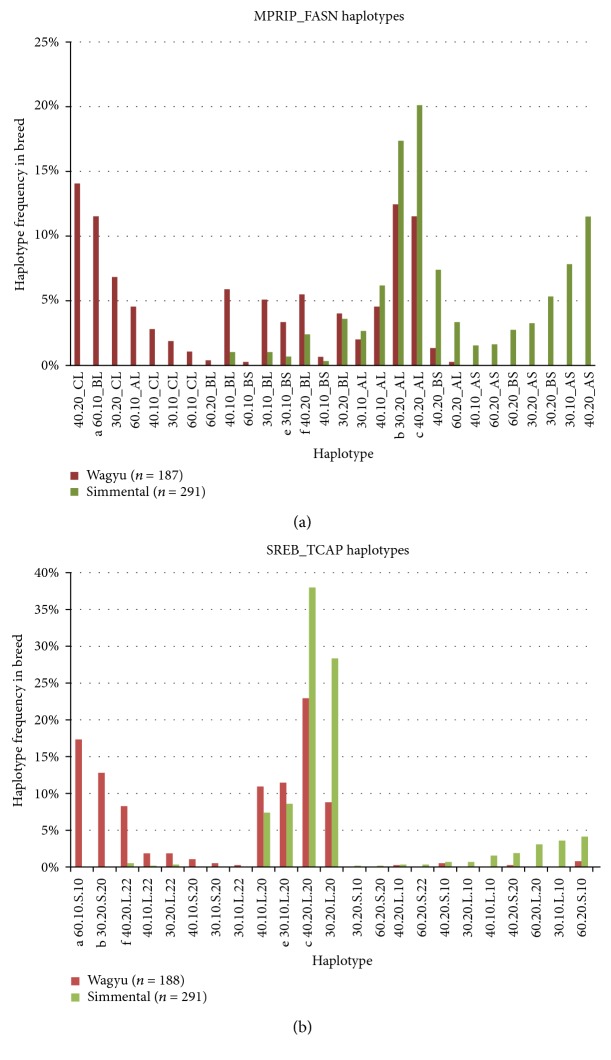
W plot comparing haplotype frequencies in Wagyu and Simmental. Adapted from [58]. (a) The haplotypes are from MPRIP to FASN (see Figure 2). Note SCT-FSN L in Wagyu and S in Simmental, also GH C in Wagyu and GH A in Simmental with GH B largely in the haplotypes common to both breeds. Haplotype designation is MPRIP.TCAP_GH SCT-FSN. (b) Haplotypes are from SREBF1 to TCAP. Haplotype designation is MPRIP.TCAP.SREB.NT5M. The four most common haplotypes of Simmental are also found at high frequency in Wagyu (and many other breeds not shown here), while two of the three most common haplotypes of Wagyu are not found in Simmental (and are not common in any other breed tested).

The 60.10.S.10 is the most common Wagyu-specific haplotype. Other haplotypes are found in all breeds (e.g., 30.20.L.20 and 40.20.L.20) and yet others are characteristic of a breed such as Simmental (e.g., 30.10.L.10). These breed differences are shown as a W plot in Figure 4.

It can be seen that the conserved ancestral haplotypes extend from SREBF1 to FASN. The breed differences are especially striking at GH and FASN. Note that Wagyu-specific haplotypes have L at FASN whereas Simmental-specific haplotypes have S (Figure 4(a)). This remarkable difference might suggest that FASN is critical, but similar breed specificity is apparent at GH. Further, an association with 60.10.S.10.B.L or 30.20.S.20.C.L in Wagyu could be misinterpreted as implicating the shared alleles SREBF1 S and FASN L which are megabases apart. Note also that the remarkable but unpredictable associations between alleles at SREBF1, GH, and FASN have the potential to explain why there has been 20 years of confusion and argument as to which particular gene is important. As also shown in Figure 4(a), the five haplotypes in the family are also present in the W plot. Three are essentially Wagyu specific whereas two are common to cattle generally implying conservation over at least thousands of generations.

By comparing the haplotypes in the red and black Wagyu, it can be seen that the 60.10 segment at M-T is shared but the SREBF1 to NTM5 (S-N) segments differ (see Figure 2). Both types of Wagyu are known for their fine marbling suggesting that M-T is crucial.

### 2.4. Haplotypes in Crossbreds

**Figure 5:**
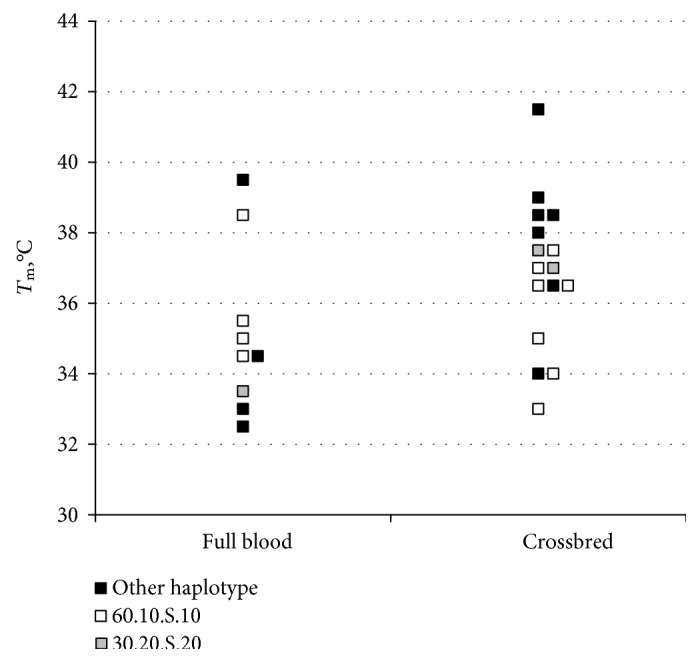
Crossbreds perform as Wagyu if they possess a Wagyu haplotype. *T*_m_ measurements from 9 full blood and 16 crossbred entries to AWA branded beef competition from 2014 and 2015. There are crossbred Wagyu with *T*_m_ as low as full bloods. Mean *T*_m_ with error bars ± 1 SEM. SREB-TCAP haplotypes. White squares indicate animals with at least one 60.10.S.10 haplotype, pale grey squares indicate animals with at least one 30.20.S.20 haplotype, and dark grey squares indicate animals with neither common Wagyu haplotype.

A key question is which Wagyu haplotypes can have an effect in crossbreds when the genetic background is foreign. Samples were taken from exhibits submitted to a steak competition intended to compare production systems rather than genetics. The results of *T*_m_ measurements are shown in Figure 5(). The full bloods are below 36°C in 7/9 as expected. Although the crossbreds, taken as a group, have higher *T*_m_, remarkably, 4/16 are below 36°C. Of the 11/16 crossbreds with *T*_m_ below 38°C, 9/11 have one or the other of two Wagyu-specific haplotypes. Thus, these two haplotypes (60.10.S.10 and 30.20.S.20) are associated with, if not directly responsible for, the dramatic fall in *T*_m_ obtained by crossing a high *T*_m_ breed with a black Wagyu which transmits one of these haplotypes. Note that these two haplotypes differ in the M-T segment but share S at SREBF1.

So as to examine the effect of haplotypes in another setting, we compared marbling scores in crossbreds differing by whether they inherited haplotypes classified as specific for either Wagyu or *Bos indicus*. As shown in Table 2, the former group had greater marbling.

### 2.5. Mapping Mechanisms to Haplotypes

It is unrealistic to expect to map single functions to ancestral haplotypes as if these were just a string of biallelic coding genes. The sequence conservation is so extensive, so complex, and yet, so poorly understood! However, it is reasonable to expect that the differences will be quantitative and that penetrance will be dependent on multiple variables. At minimum, there will be multiple *cis-* and *trans*-interactions between the various polymorphisms. It follows that we expect to find associations at multiple markers. The results given above appear to implicate sequences around S and within the M-T segment of Wagyu haplotypes irrespective of effects marked by GH and FASN (see Figure 2).

In order to evaluate such conclusions and so as to assess other markers and haplotypes, we have taken advantage of the availability of two very different data sets:

Mayura**—**fixed environment including 300 DOF, 100% black Wagyu, permitting examination of genetic differences within black Wagyu.

Melaleuka**—**fixed environment but variable DOF and breed, permitting examination of all haplotypes, Wagyu haplotypes within crossbreds and effects on the *rate* of marbling

Table 3 gives some of the results of the Mayura set. As already shown, see Figure 4, this is the set which revealed the heritability of *T*_m_ confirming its value in dissecting complex genetics. For convenience, we show a cut-off of 37°C, giving approximately 50% in the above and below groups. By comparing alleles at four of the loci shown in Figure 2, it is clear that the TCAP 20 allele is associated with lower *T*_m_. The MPRIP 60 is associated with higher *T*_m_. Although SREBF1 S is a marker for low *T*_m_ in some earlier studies and patents, here we find the opposite. At the NT5M locus, the 22 allele is associated with low *T*_m_.

The inconsistencies between past and present results and between locus and haplotype analyses can be illustrated as follows. The prototypic Wagyu ancestral sequence, 60.10.S10, can be recognized by the 60.10.S.10 combination, but these alleles, taken individually, are *not* haplospecific. As shown in Figure 2, each is found on more than one haplotype.

When the alleles are examined without regard to whether they are carried by the ancestral haplotype, *T*_m_ would be higher at MPRIP, TCAP, SREB-P, and NT5M (see Table 3). However, as shown in Figure 5, the 60.10.S.10 ancestral haplotype actually lowers the *T*_m_ of crossbreds. Thus, there may be multiple *cis*- and *trans*-interactions contributing to the effect of a particular haplotype.

**Figure 6:**
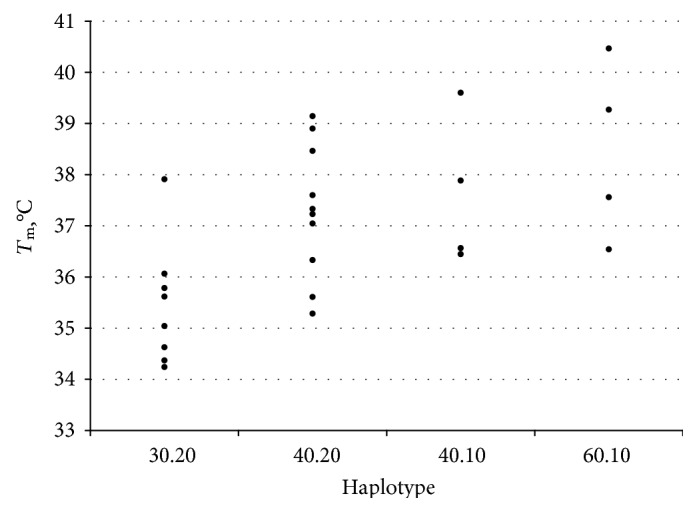
Dot plots of *T*_m_ values in Wagyu 300 ± 20 DOF in animals homozygous for the 5.24 Mb region MPRIP-TCAP. Wagyu cattle bred on different Australian farms (Goorambat, Irongate Wagyu, Mayura, Rosevale, and Peppermint Grove) were fed for 300 days at Mayura Station, South Australia, and beef/fat samples were assayed for C19 haplotypes and fat melting point *T*_m_ as indicated in Materials and Methods. Each dot point represents a different homozygote. Homozygous haplotypes for MPRIP-TCAP region only. Student's *t*-test for 30.20 versus 40.20 yields *P* = 0.012.

As a further approach to address the complexity, we asked whether there is any influence of *trans*-interaction or homozygosity. Since it would be necessary to study many hundreds of thousands of individuals to obtain sufficient homozygotes of even the most common haplotypes, we chose to examine the least polymorphic loci such as TCAP with only two alleles: 10 and 20. In the Mayura long-fed Wagyu data set, all 8 with *T*_m_ below 36°C are 20,20 homozygotes (see Figure 6). Using 37°C as a cut-off, 60% of 20,20 are lower compared with only 33% of the 10,10 homozygotes.

The effect of TCAP homozygosity is clear in a genetically very diverse group fed for 450 days. Only subcutaneous fat from over the rump was available. Although not comparable to other samples reported above, TCAP 10,10 homozygotes have higher *T*_m_ than 20,20 homozygotes (Table 4).

**Figure 7:**
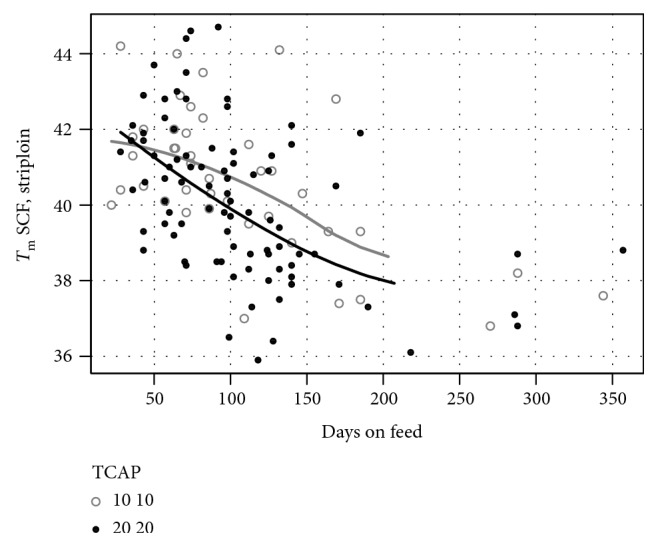
TCAP 20 homozygotes achieve low *T*_m_ with less days on feed (Melaleuka crossbreds). TCAP 20 homozygotes (filled circles) achieve low *T*_m_ with less days on feed. Hollow circles show TCAP 10 homozygotes. The smoothed lines were calculated in R with a span of 100. *T*_m_ measured on various European and Japanese breeds and crossbreeds including Simmental, Gelbvieh, black Wagyu, and red Wagyu. Subcutaneous fat samples were taken from the rump and front ends of striploins, with the *T*_m_ reported as the average of the two samples. Striploins were DNA tested to confirm a match to the animal sent to abattoir. 42 samples from TCAP 10 homozygotes and 87 samples from TCAP 20 homozygotes are shown. *T*_m_ measurements of 105 samples from TCAP 10,20 heterozygotes have been excluded from the graph for simplicity.

**Figure 8:**
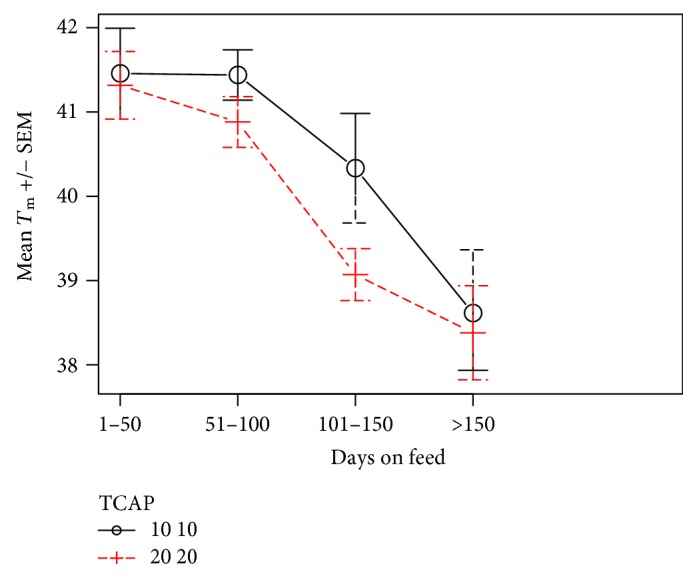
TCAP 20 animals have significantly lower *T*_m_ for 50–150 days on feed. The presence of homozygous 20 TCAP alleles makes the decrease in *T*_m_ occur at less days on feed. The initial *T*_m_ is similar between the two groups. The *T*_m_ in both groups appears to reach a minimum level with long feeding that is not affected by the TCAP allele.

So as to address the issue in a very different data set, we analysed the Melaleuka Euro and crossbred cattle and found similar results. As shown in Figure 7(), TCAP 10,10 and 20,20 homozygotes diverge after 50–100 DOF. After 100 DOF, 9 of 12 with TCAP 10,10 remain above 39°C compared with 3 of 12 with TCAP 20,20 (*P* < 0.05). The 20,20 homozygotes assume a faster trajectory to healthier fat indicating a quantitative regulatory effect (Figure 8()).

**Figure 9:**
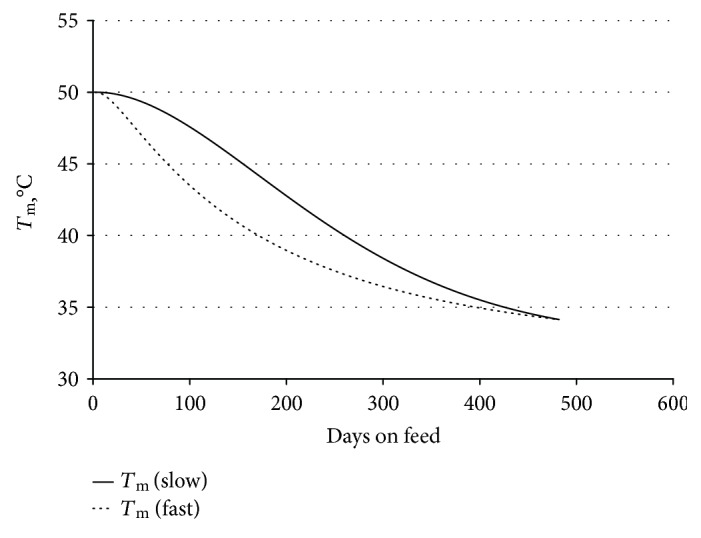
Match to desaturation model of fat melting point. The *T*_m_ decrease shown in Figure 7 can be described by the following mathematical model of fat production and desaturation. De novo fat production is controlled by FASN, in response to energy balance. This fat starts saturated. It is then desaturated by the SCD enzyme. The total fat present (*F*) can be described by *dF*/*dt* = *a*, where *a* is the rate of de novo fat production. The amount of unsaturated fat (*U*) increases in proportion to the amount of desaturation enzyme *E* as *dU*/*dt* = *E*. In this model, the amount of enzyme available is increased in response to the amount of saturated fat present, and also decays. The amount of enzyme is described by *dE*/*dt* = *bS* − *cE*, where *b* and *c* are constant control parameters and *S* = *F* − *U* is the amount of fat that remains unsaturated. The melting point decreases with increasing proportion of unsaturated fat. *T*_m_ decreases in proportion to the amount of unsaturated fat, *T*_m_ = *T*_Δ_ and *S*/*F* + *T*_U_. The melting point curves resulting from this model have an initial high *T*_m_ decreasing slowly at first and then more rapidly as the desaturation enzyme builds up. This figure shows how the model reacts to parameter changes. The fast enzyme response with parameters *b* = .001 and *c* = .1 looks similar to the *T*_m_ curve shown in Figure 8, TCAP 20 homozygotes. A slower enzyme response has parameters of *b* = .0001 and *c* = .01 is more similar to the *T*_m_ curve for TCAP 10 homozygotes. TCAP is already known to be involved in pathways regulating the production of the SCD enzyme, so it influencing the T_m_ in this way is not surprising.

A similar pattern is obtained when a model is constructed based on the slow induction of SCD (Figure 9()).

## 3. Discussion

The history of Japanese Wagyu or “Kobe” beef is intriguing. According to legend [3], Wagyu cattle were used for heavy burden although fed only with rice husks, suggesting that they may have been selected for efficient food conversion combined with an ability to utilise fatty acids as a direct energy source. Much later, after the eating of meat was permitted, selection (according to the Shogun's preferences) must have led to enhanced flavour as found with fine marbling and low melting temperature intramuscular fat. Another consequence has been a tendency to muscle weakness attributed to the replacement of myocytes with adipocytes. Whatever the selection criteria, Wagyu have conserved the capacity to marble from their remote North Asian ancestors and seemingly still do so after crossing with diverse breeds now found worldwide. This history suggested that there must be highly conserved and penetrant haplotypes rather than recent random mutations.

The present study began with the seemingly simple aim of improving the healthiness of beef by crossing elite Japanese Wagyu with composite breeds able to survive in harsh conditions. The literature suggests that transfer of a Wagyu gene might improve the fat content thereby providing cholesterol-lowering, statin-sparing, tasty beef at reasonable cost. Experience has shown that improvement can occur but inconsistently and only after expensive grain-feeding thereby restricting worldwide access to potential benefits. Our interpretation is that the outcome depends on the particular mix of conserved haplotypes which exert quantitative control over all the multiple functions which shift the balance from muscle to lipid and especially MUFA.

As shown elsewhere [60], it was necessary to develop a simple method of measuring the amount of beneficial fat. It transpires that heritability of the preferred fat can be demonstrated with *T*_m_ but not the standard visual method of quantifying marbling. Hitherto, therefore, selection of sires must have suffered. This is important because, using *T*_m_, the sire effect is evident within one generation, meaning that success or failure can occur rapidly, leading to the remarkable scatter as demonstrated in Figure 2. Fortunately, *T*_m_ measurements should lead to progressive improvement.

Given accurate and precise measurement of the endpoints, we have used four data sets.

Samples from a tasting competition reveal that some crossbreds can be as desirable as the most elite Wagyu. Here, we offer a strategy based on the observation that transmission of a certain Wagyu haplotype, such as 60.10.S.10, may dramatically improve quality. If confirmed, a homozygous bull will benefit all progeny and remove some of the existing inconsistency and expense. In an ongoing multicentre study, we hope to be able to evaluate the benefits of sires which are homozygous for 30.20.S.20 and 60.10.S.10 in their anticipated order of benefit. However, because of relatively low haplotype frequencies of say 0.05, the natural frequencies of homozygotes will be less than 0.0025, meaning that the value of such homozygotes will only be clear if all bulls are haplotyped. The solution to the lack of homozygotes for use today is being addressed through artificial insemination and embryo transfer. In the meanwhile, we are developing some hypotheses which recognize that evaluating all combinations of multimegabase haplotypes (as diplotypes) is impractical. The *n* values required for the comparisons are greater than the number of cattle alive today.

One of these hypotheses arose from the ability to compare two totally different data sets. The Mayura set standardizes essentially all variables, other than the genetic differences between Wagyu. The Melaleuka set uses *T*_m_/DOF as a measure to compare crossbreds.

Both show a remarkable effect of TCAP 10,10 versus 20,20. It should be emphasized that differences at TCAP itself cannot explain all findings such as the superiority of Wagyu over other breeds and the benefit of 60.10.S.10 in crossbreds.

Rather, taken together, the results argue for the importance of ancestral haplotypes rather than individual loci. This is illustrated in Figure 4(). The alleles at GH and FASN are different depending upon the megabase haplotype, meaning that effects attributed to these individual alleles could reflect sequences more than 10 megabases away. Therefore, it is important to define the megabase haplotypes before sequencing or SNP analyses. No doubt, this explains why progress has been slow. Genomic analysis of polygenic traits like QTLs must consider the implications of synteny and the potential importance of paralogy, duplication, copy number, and retroviral regulators to list a few mechanisms [2, 3, 30, 31, 55]. As proposed here, duplication and copy number are more relevant than coding region differences; so too is regulation over extensive distances. Thus, SREBF1 is a master regulator which controls gene expression within the SREBF1 to FASN region via targeting transcriptional repressors of MPRIP, TCAP, and other muscle–related genes (BECN1, CACNB1, MYH2, MYH3, and MYH13); one of these repressors BHLHB 2 maps near FASN [55]. Note also that muscular dystrophy, limb girdle type 2G is associated in humans with TCAP polymorphism (OMIM#604488) and the histopathology resembles marbling in some respects. Lipid accumulation has been observed in limb girdle muscular dystrophy [61]. Another unexpected recent finding is the location of UTS2R which is associated with development of type II diabetes and fat deposition [44].

The balance between myogenesis and adipogenesis may be regulated in part by the TCAP-myostatin pathway in Wagyu as first suggested by Shibata et al. [62].

We conclude that genomic differences regulate the amount of MUFA such as oleic acid in beef and determine the *rate* at which *T*_m_ falls with DOF. We regard this finding as indicating, possibly for the first time in the setting of genomics, that the genetic dissection confirms the expectation that the important differences are conserved and quantitative, rather than qualitative, in keeping with our earlier studies on regulation of immunoglobulin and antibodies by MHC ancestral haplotypes [2, 11–13].

Genomic markers for favorable rates of marbling will be of practical as well as theoretical interest. Reducing the time and cost on feed brings the potential to provide healthy beef to a wider sector of the world's population with the secondary benefits of reducing cardiovascular disease and the billions of dollars spent on cholesterol lowering statins.

One of the most important lessons from the present study is that a haplotype sequence of multiple genes and regulators can have complex associations and confounding effects. The 60.10.S.10 haplotypes is Wagyu specific, beneficial in upgrading non-Wagyu in the tasting competition, directly or indirectly associated with low *T*_m_ but only the 3rd or 4th best in the ranking of the Wagyu haplotypes. We believe that further data will show that, as in the case of the human MHC, diplotypes and their quantitative *cis*- and *trans*-interactions will be critical in explaining apparent contradictions.

In conclusion, we recommend combining an accurate measurement of the phenotype with a genomic description of the genotype allowing identification of megabase ancestral haplotypes.

## 4. Materials and Methods

### 4.1. Full-Blood Wagyu with Identified Sires

Two cohorts of Wagyu steers (*n* = 128) and heifers (*n* = 6) were fed for approximately 300 ± 20 days on a proprietary ration within a commercial feedlot. One-gram samples of meat and intramuscular fat were taken from each carcass between the 10th and 11th rib. AUS-MEAT marbling score (MS) was scored between the 10th and 11th rib, with an average of 7.6 and a range from 2 to 11. Steers chosen for the sire comparison had their paternity confirmed via DNA testing [55].

### 4.2. Branded Beef Competition

Samples were obtained from 9 full blood, 16 crossbred, and 4 grass-fed entries to the Australian Wagyu Association's “Branded Beef Competition” from 2014 to 2015. Crossbred entries were F1, F2, F3, and F4 Wagyu. Feed composition and days on feed varied, but all entries had more than 25% intramuscular fat, as assessed by camera image analysis [64].

### 4.3. Short-Fed European and Crossbreds

The herd at Melaleuka Stud has a variety of European breeds including Simmental, Gelbvieh, and Angus. This herd was selected to produce high-quality beef on pasture, finished within 2 to 4 months of supplemental feeding. Black Wagyu have been introduced into the herd as full blood or pure bred and for crossing with some of the European breeds. Those designated 50% are the F1 progeny of a mating between Wagyu and European, whereas 25% and 75% are the second cross progeny of F1s mated with European and Wagyu, respectively.

Akaushi sires have recently been used over some European breed cows with European breed bulls continuing to be used over the remaining cows.

Calves stay on milk until 4 months of age when they are weaned, and male calves are castrated. After weaning, they continue grazing Kikuyu and Ryegrass pasture until they reach 300 kg. Their feed is then supplemented with 9 mm EasyBeef pellets (Milne Feeds, Perth, Australia) ad libitum. The main ingredients of the EasyBeef pellets are lupins, barley, oats, wheat, and triticale. The nutritional composition, based on dry matter, is crude protein (min) 14.5%, metabolizable energy (est.) 11.0 MJ/kg, crude fiber (max) 20.0%, urea (max) 1.5%, and monensin 26.6 ppm.

The feeders are considered ready for slaughter when they reach a weight of 400 kg and are slaughtered to match demand. Some animals were kept on feed longer to test the effect of increased feeding on *T*_m_ and meat quality. The average live weight at slaughter for animals in this study was 461 kg, average age at slaughter was 15.4 months (range 8 to 23), and the average days on feed was 104 days (range 17 to 288). Body numbers from abattoirs were matched to farm records and pedigrees via their RFID tags, where possible identity was confirmed by in-house proprietary DNA testing [56].

Subcutaneous fat samples from the sirloin of these cattle were collected after boning and wet aging.

### 4.4. Long-Fed Wagyu Crossbreeds with *Bos indicus* Ancestry

Fifty samples of subcutaneous fat were taken from the rump of crossbred Wagyu steers fed for 450 days in a commercial feedlot. Samples were collected from the abattoir and frozen immediately. DNA was extracted and tested for MPRIP markers, and triglycerides which separated from the adipose tissue during DNA extraction were tested for *T*_m_. Wagyu ancestry ranged from 48% to 96% with the majority being F1 and average Wagyu ancestry at 57%. 19 of the 50 animals had some *Bos indicus* content. Other dam breeds included Shorthorn and Angus.

### 4.5. DNA Extraction and C19 Haplotype Testing

Genomic DNA was extracted from all meat samples using the standard salting out method. Alleles of SREB, NT5M, MPRIP, TCAP, and GH markers were determined by PCR and capillary electrophoresis using the primers and method described in Lecomte et al. [55]. Alleles of FASN were determined using the primer and method described in Williamson et al. [56]. The FASN marker is more correctly known as SCT-FSN since the present marker is adjacent to the coding region for FASN, within the segmental duplication containing SECTM1. Haplotypes in heterozygous individuals were determined from segregation or alleles in related animals. The W plots were based on haplotype frequencies measured in Australian herds of registered Wagyu.

## Supplementary Material

Supplementary Figure 1: The three dotplots show the similarity between regions of the bovine chromsome 19 on the horizontal axis and the human chromosome 17 on the vertical axis. Diagonal lines from top left to bottom right show homologous regions, while diagonals in the opposite regions show homologous regions that have been through a process of inversion. Colour shading matches to the regions shown in Figure 1.

## Figures and Tables

**Table 1:** Candidate genes at c19 35 Mb to 55 Mb with reported associations with fatty acid composition.

Gene	Reference	Effect on intramuscular fatty acid composition in beef cattle muscle tissues
SREBF1	[37]	In Japanese black cattle: S allele associated higher MUFA and 1.6°C lower *T*_m_ of IMF.
	[38]	In Korean Hanwoo cattle: SS alleles IMF stearic acid (C18:0) lower than LL (*P* < 0.05) but linoleic and PUFA contents higher in SS compared to LL (*P* < 0.05).
	[39]	In Japanese black cattle: no associations with fatty acid composition or meat yield traits.
	[40]	Canadian crossbred steers S/L polymorphism associated with 9c C17:1 (*P* = 0.013).
	[41]	Simmental bulls, snow dragon black: LL higher palmitic acid (C16:1), triglycerides, and C16 index but lower stearic acid (C18:0) and SFA compared with the LS genotype (*P* < 0.05).
		
TCAP	[42]	In Korean Hanwoo cattle: a 6 bp Leu-Gln deletion as well as SNP in intron 1 associates with marbling score (*P* = 0.02, *P* = 0.003, resp.).
		
GH	[43]	In Japanese black cattle: A allele associated low *T*_m_ fat; allele B gave higher % C18:1 *n* = 9 IMF (*P* < 0.05); allele C gave higher C18:1 MUFA, higher USFA & (*P* < 0.05). Allele C also gave lower % saturated fatty acid (SFA), a higher MUFA/SFA ratio, and lower *T*_m_ of fat (*P* < 0.01).
	[39]	GH L127V polymorphism (A/B Ardiyanti et al. 2009) claimed association with IMF fatty acid composition, but not as strong as FASN, SCD or SREBF1.
		
UTS2R	[44]	Japanese black 7 Holstein cattle: Reported differential association between breeds with marbling and non-synonymous SNP in coding region.
		
FASN	[45]	Angus bulls: SNP coding region g.17924A>G (as GG genotype) associated FA composition – lower myristic acid (C14:0 *P* < 0.00001), palmitic acid (C16:0 *P* < 0.05) and total saturated FA (*P* < 0.01) in total lipids and TAG than g.17924AA genotype.
	[46]	Japanese black x Limousin F2 exon 34 SNPs (g.16024A>G (T1950A), g.16039T>C (W1955R). TW changes together increase C18:0, C18:1 content, increase MUFA:SFA ratio.
	[38]	Korean Hanwoo cattle: g17924G>A SNP as GG genotype with higher Oleic Acid (C18:1) palmitic acid (C16:0).
	[48]	GWAS Oleic Acid signal g.16024A>G.
	[49]	GWAS reveal significant SNP signals associated FA composition near FASN gene. Confirms Uemoto et al. 2011.
	[39]	Japanese black cattle: significant effects on C14:0, C14:1, C18:0, oleic acid C18:1 and MUFA (all *P* < 0.001).
	[47]	Hanwoo Korean cattle: all 5 known exonic SNPs associated with FA composition IMF and marbling score.
	[50]	Angus cattle: GWAS 51st Mb window on c19 harboring FASN associated with FA composition of IMF.
	[51]	Japanese black cattle: seven known SNPs. Promoter g.841G>C-improved FA composition IMF.
	[52]	Fleckvieh bulls:known FASN SNP significant associations C14:0, C16:0, and C18:1n-9 in IMF.
	[53]	Canadian study: GWAS, same bead chip as Uemoto et al. 2011 “markers have large effects near FASN and SCD.”
	[54]	Angus, Hereford, Limousin crossbreds: g.16024A>G SNP. AG genotype with higher IMF than GG genotype.

SREBF1—transcription factor that regulates gene expression levels of stearoyl-CoA desaturase (SCD) leading predominantly to monounsaturated fatty acid (MUFA) oleic acid C18:1n = 9 in intramuscular fat (IMF); TCAP—titin-cap or Telethonin, interacts with titin-cap structure and regulates, by inhibition, myostatin hormone secretion; GH1—growth hormone; UTS2R—urotensin 2 receptor; FASN—fatty acid synthetase.

**Table 2:** Higher marble score in F1 and F2 Wagyu with Wagyu-specific haplotypes compared to *Bos indicus*-specific haplotypes.

	Average MS	N	SEM
*Bos indicus* haplotypes	4.2	6	0.54
Wagyu haplotypes	5.3	22	0.22

Crossbred and pure bred Wagyu fed for 450 days marbling assessed visually between the 10th and 11th rib, using the AUS-Meat marble scale. Breeds of dams include Brahman, Shorthorn, and Angus. Haplotypes were determined for SREB to TCAP markers by a combination of pedigree analysis, homozygosity, and breed-based haplotype frequencies. Haplotypes classified as Wagyu specific were 60.10.S.10, 30.20.S.20, and 30.10.S.20 with frequencies in Wagyu shown in Figure 4(b). Haplotypes classified as *Bos indicus* specific were those with MRIP > 60 or NT5M > 22. For simplicity, animals with neither a *Bos indicus*-specific nor a Wagyu-specific haplotype have been excluded from the table.

**(a):** 

		SREBF1 alleles		
*T*_m_		S	L	Total
<37°C		41	89	130
≥37°C		52	82	134
Total		93	171	264

Fisher exact test statistic 0.25. Not significant at *P* < 0.05.

**(b):** 

	NT5M alleles			
*T*_m_	10	20	22	Total
<37°C	19	84	27	130
≥37°C	32	82	20	134
Total	51	166	47	264

Chi-squared statistic is 4.32. The *P* value is 0.1. Not significant at *P* < 0.05.

**(c):** 

	MPRIP alleles			
*T*_m_	30	40	60	Total
<37°C	48	60	18	126
≥37°C	34	66	32	132
Total	82	126	50	258

Chi-squared statistic is 6.5. The *P* value is 0.04. Result is significant at *P* < 0.05. The total is lower as some animals with *T*_m_ were not typed for MPRIP allele.

**(d):** 

	TCAP alleles		
*T*_m_	10	20	Total
<37°C	55	75	130
≥37°C	74	60	134
Total	129	135	264

Fisher's exact test statistic is 0.04. The result is significant at *P* < 0.05.

**Table 4:** In crossbred and pure bred Wagyu fed for 450 days, those with homozygous TCAP 10 had higher *T*_m_ of the subcutaneous fat over the rump. Breeds of dams include Brahman, Shorthorn, and Angus. For simplicity, animals heterozygous at TCAP have been excluded from the table.

TCAP genotype	Subcutaneous rump *T*_m_	N	SEM
10 10	35.7	4	0.46
20 20	33.2	22	0.75
