# Supplementary figures and images for: Haplotypes for Type, Degree, and Rate of Marbling in Cattle Are Syntenic with Human Muscular Dystrophy
Source: Int J Genomics. 2017 Aug 17;2017:6532837. doi: 10.1155/2017/6532837 (PMC5585636; doi:10.1155/2017/6532837)

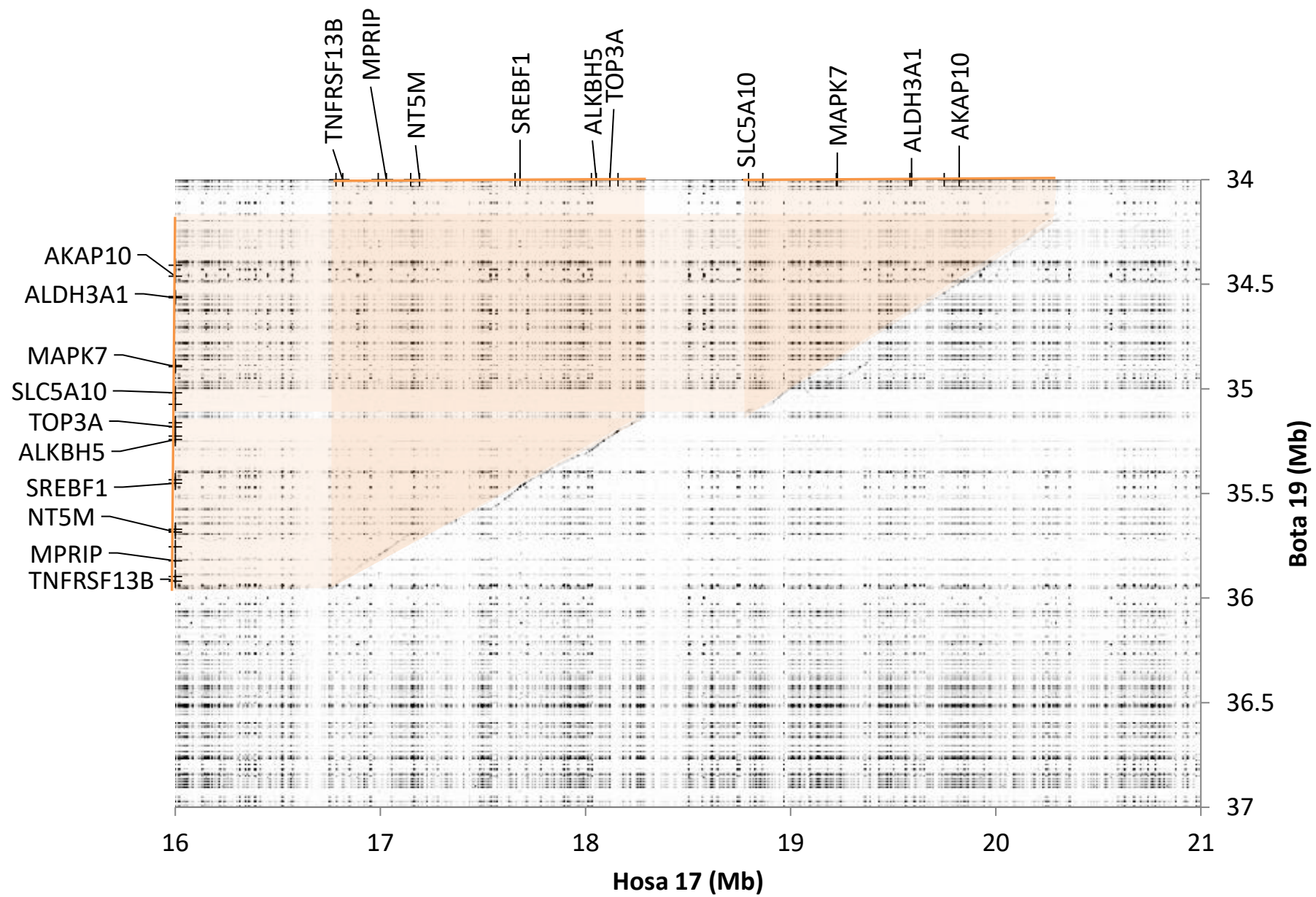

Supplement: Supplementary file 1 — Supplementary Figure 1: The three dotplots show the similarity between regions of the bovine chromsome 19 on the horizontal axis and the human chromosome 17 on the vertical axis. Diagonal lines from top left to bottom right show homologous regions, while diagonals in the opposite regions show homologous regions that have been through a process of inversion. Colour shading matches to the regions shown in Figure 1. [file 6532837.f1.pdf]

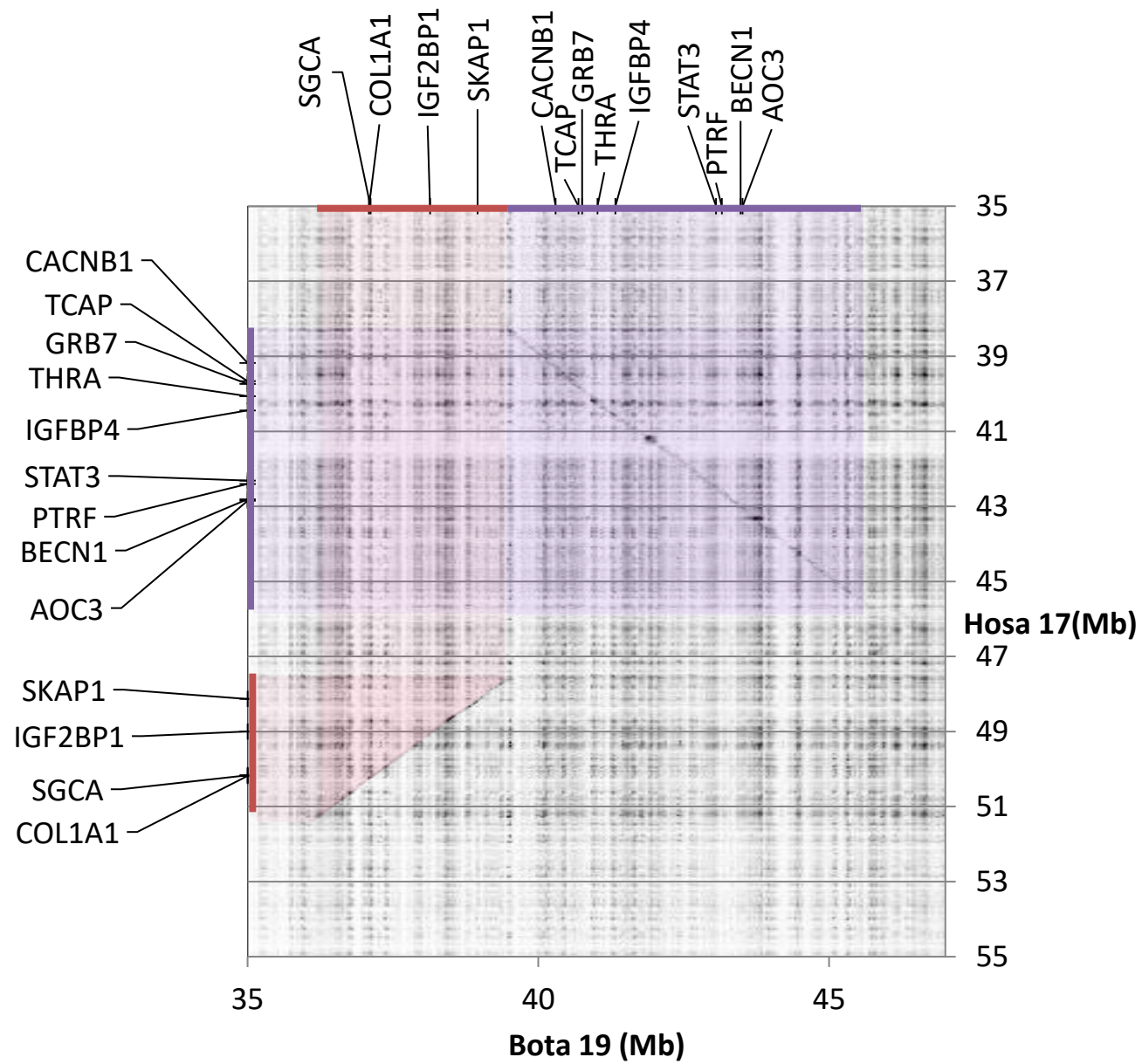

Supplement: Supplementary file 2 [file 6532837.f2.pdf]

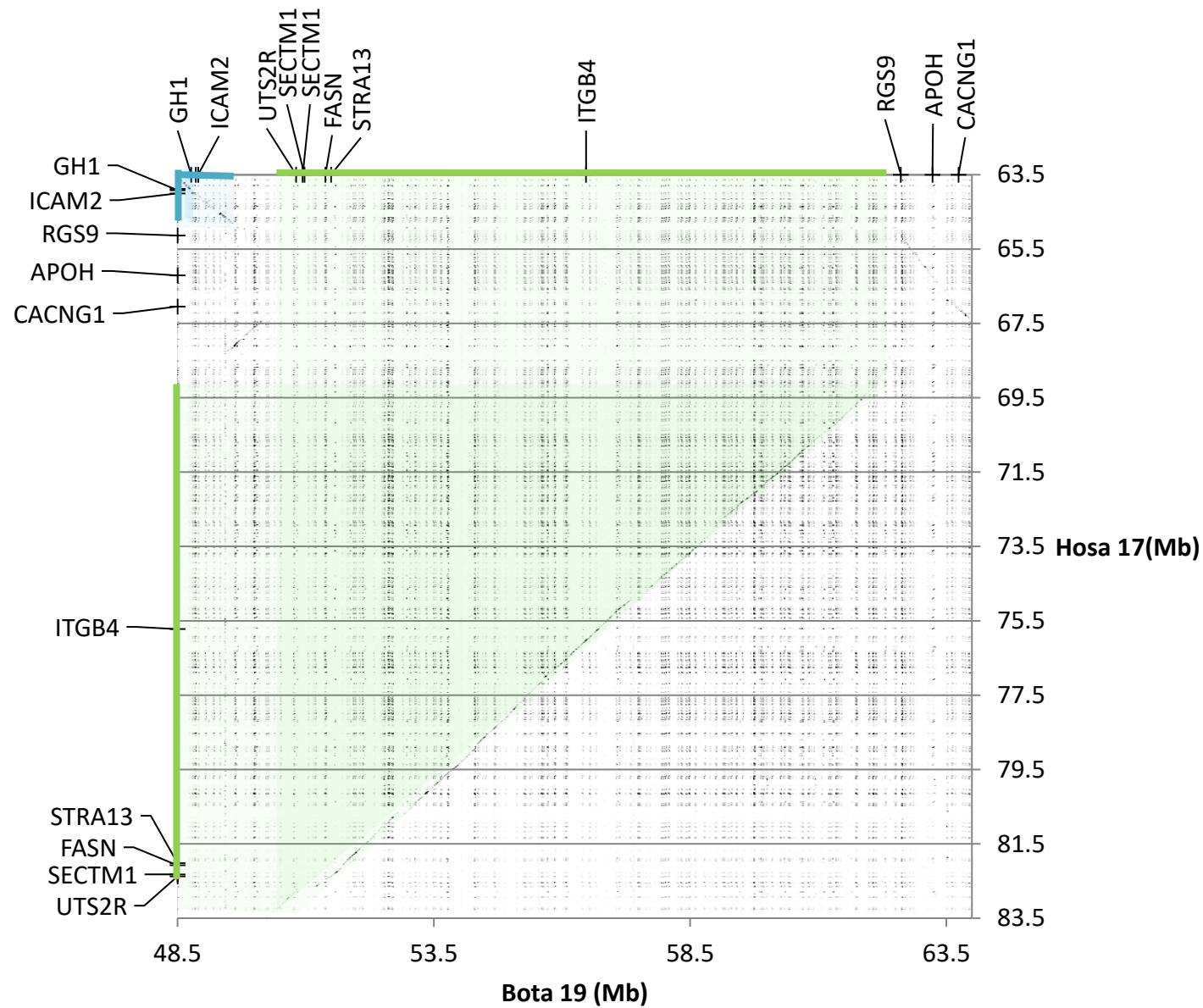

Supplement: Supplementary file 3 [file 6532837.f3.pdf]
